# Statin use and outcomes of oncological treatment for castration-resistant prostate cancer

**DOI:** 10.1038/s41598-023-45958-8

**Published:** 2023-11-01

**Authors:** A. I. Peltomaa, K. Talala, K. Taari, T. L. J. Tammela, A. Auvinen, T. J. Murtola

**Affiliations:** 1 Faculty of Medicine and Health Technology, Tampere University and Tampere University Hospital, Teiskontie 35, 33521 Tampere, Finland; 2 Department of Radiology, HUS Medical Imaging Center, University of Helsinki and Helsinki University Hospital, Helsinki, Finland; 3 Finnish Cancer Registry, Helsinki, Finland; 4 Department of Urology, University of Helsinki and Helsinki University Hospital, Helsinki, Finland; 5 Department of Urology, TAYS Cancer Center, Tampere, Finland; 6 Faculty of Social Sciences, Tampere University, Tampere, Finland

**Keywords:** Cancer prevention, Urological cancer, Cancer, Cancer epidemiology

## Abstract

To compare the effect of statin use in relation to castration-resistant prostate cancer (CRPC) treatment, we assessed the risk of ADT-treated PCa-patients to initiate CRPC treatment by statin use and the outcomes of CRPC treatment by statin use. Our study cohort consisted of 1169 men who participated in the Finnish Randomized Study of Screening for Prostate Cancer (FinRSPC) and initiated androgen deprivation therapy (ADT) during the follow-up (1996–2017). Statin use was associated with slightly decreased risk of initiating CRPC treatment (HR 0.68; 95% CI 0.47–0.97) with a 5.7 years’ median follow-up until CRPC for non-users and 7.5 years for statin users. The risk of discontinuation of first or second line CRPC treatment due to inefficacy was not modified by statin use and the results remained similar in subgroup analysis assessing separately patients treated with taxans or androgen receptor signaling inhibitors. We observed an inverse association between statin use and the risk of initiation of the CRPC treatment. No beneficial risk modification by statin use during CRPC treatment was observed. These results suggest that statins might be beneficial during hormone-sensitive phase but not in the later phases of prostate cancer treatment.

## Introduction

Statins lower blood cholesterol levels and decrease morbidity and mortality in cardiovascular diseases, especially in secondary prevention^1^. The beneficial effect of statins seems, however, not to be limited to cardiovascular prevention. Several studies have found statins to be linked to either improved prostate cancer mortality or progression-free survival^2–6^. However, results on statins’ effect on prostate cancer mortality or risk of biochemical recurrence have been partly conflicting. Hence, it is still unclear if the possible benefit of statins is limited to a subgroup of cancer patients e.g. those receiving specific cancer treatment^7–9^. Especially, prostate cancer patients on androgen deprivation therapy have been consistently reported to benefit from concurrent statin use^10–13^.

In vitro studies have proposed possible mechanisms that may explain potential additive effects of statin use in combination with androgen deprivation therapy, chemotherapy or androgen receptor signaling inhibitors. Harshman et al. showed statins to competitively reduce uptake of dehydroepiandrosterone sulfate (DHEAS) by prostate cancer cells suggesting a plausible mechanism to reduce supply of DHEAS and act in synergism with ADT^12^. Raittinen et al.^14^ compared atorvastatin to placebo and reported atorvastatin to induce adrenal androgen downshift possibly providing a novel pathway reducing androgen concentration in prostate cancer patients. Hu et al.^15^ found that statins have synergistic effect with abiraterone on neuroblastoma progression in vitro and in vivo. Our previous report showed an additive inhibitory effect of statins on prostate cancer cells in combination with enzalutamide^16^. Some studies have also demonstrated growth inhibition or induction of apoptosis in prostate cancer cells for statins in combination with docetaxel^17–20^.

Several epidemiological studies and at least one meta-analysis have reported improved overall and cancer-specific survival in prostate cancer patients treated with statins plus androgen deprivation therapy^21^. However, the possible modifying effect of statin use on prognosis of castration-resistant prostate cancer patients treated with docetaxel, enzalutamide or abiraterone still needs further evaluation. Some studies have shown an additive effect of statins combined with either abiraterone or abiraterone/enzalutamide^22–24^, but studies assessing statins’ effect on PCa patients treated with docetaxel are scarce.

We assessed the risk of developing castration-resistant prostate cancer (CRPC), risk of initiation of first- or second-line CRPC treatment and discontinuation of CRPC treatment due to inefficacy in a population-based cohort of men receiving androgen deprivation therapy for prostate cancer.

## Methods

### Study cohort

The Finnish Randomized Study of Screening for Prostate Cancer (FinRSPC) is a randomized population-based trial assessing the effect of systematic screening with prostate-specific antigen on prostate cancer mortality. The trial population consisted of all men aged 55, 59, 63 or 67 at baseline residing in Helsinki and Tampere metropolitan areas in Finland. We identified all participants of FinRSPC from Tampere metropolitan area diagnosed with prostate cancer and initiating androgen deprivation therapy during 1996–2017. Those 1169 men formed the study cohort for the present study. The follow-up started at the initiation of androgen deprivation therapy and continued until death, emigration from Finland or 31 December 2017, whichever occurred first.

Statistics Finland registers all deaths in Finland. Patients with prostate cancer (ICD-10 C61) as a primary cause of death were defined as prostate cancer deaths. In addition, all other causes of deaths were acquired from the database. Statistics Finland gave permission for using the cause of death data (TK/3536/07.03.00/2021).

The FinRSPC study protocol (approved by the Ethics Port of Pirkanmaa Hospital; decision number ETL95077) has been described comprehensively previously and informed consent was obtained from all FinRSPC participants^25^. The Finnish Institute for Health and Welfare has approved this study protocol and all methods were carried out in accordance to relevant guidelines.

### Information on medication use and treatment

**Figure 1:**
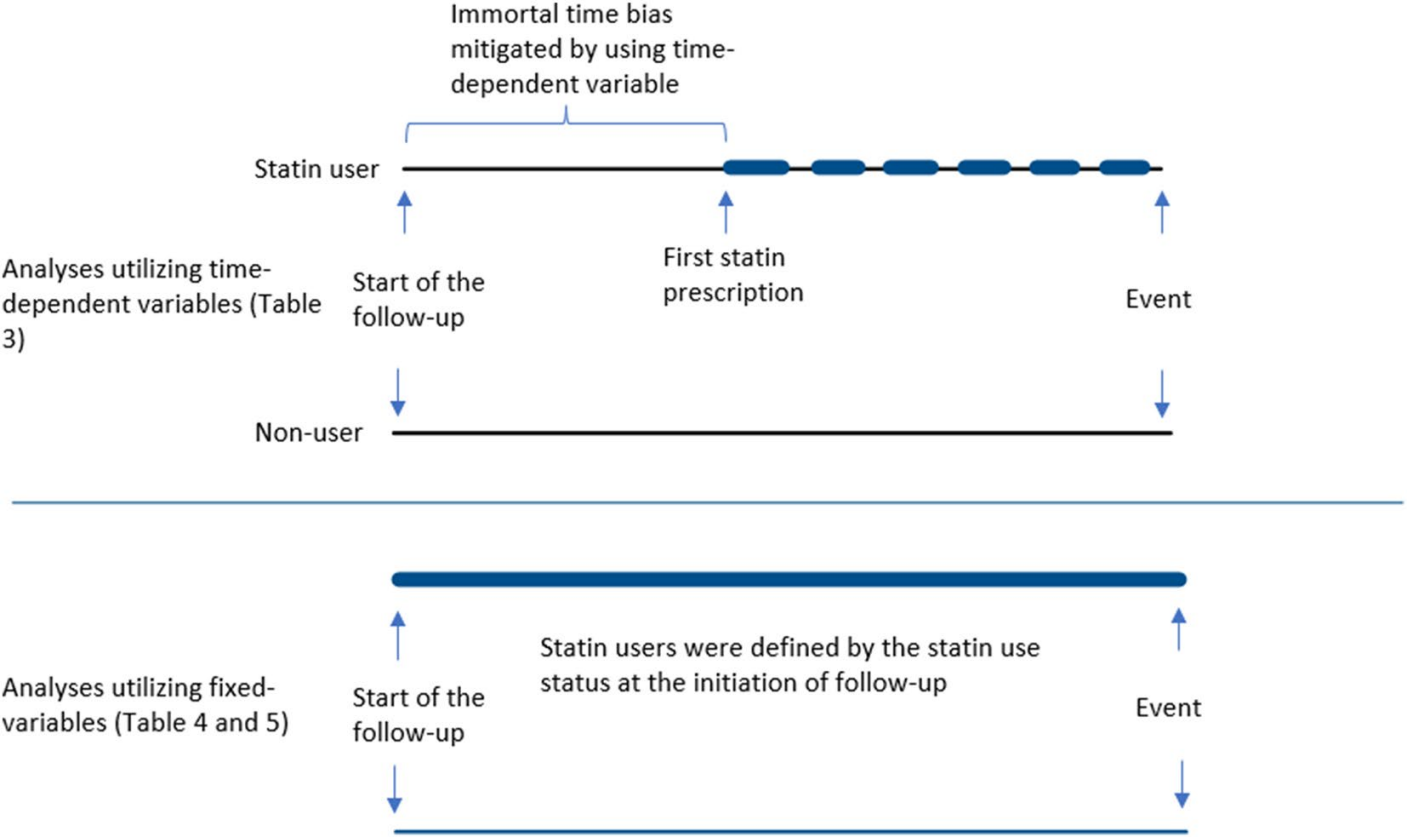
Illustration of statin exposure in analyses utilizing, (**a**) time-dependent statin use variables, used in Table 3 and (**b**) fixed statin use variables, used in Tables 4 and 5.

The study cohort was linked to the National Prescription Database maintained by Social Insurance Institute of Finland (SII) to obtain information on statin purchases. SII provides reimbursements for purchases of physician-prescribed medications in Finland and registers nearly all drug purchases (over-the-counter purchases not included). The reimbursement system has been described in detail previously^2^. Information on the amount, dosage, generic name, and purchase dates was obtained for all statin purchases. In analyses utilizing ‘any statin use’ each year with recorded statin purchases was considered as a year of usage. The mg amount of different statins was standardized by dividing the total mg amount of statins with the amount corresponding to a Defined Daily Dose (DDD) defined by the World Health Organization^26^. Intensity of statin use (average DDDs/year) was calculated by dividing cumulative DDDs with number of years with statin usage. Statin use status was allowed to change on a yearly basis. Dichotomous statin use variables (any/none) and intensity variables were updated for each follow-up year based on recorded purchases. After first reimbursed drug purchase, status remained as ever user even when purchases stopped, to limit bias related to the tendency to discontinue statins during the final stages of cancer. Statin exposure in different analyses is illustrated in Fig. 1.

The cohort was also linked to the database of Care Register for Health Care (Hilmo) maintained by the National Institute for Health and Welfare to obtain diagnoses from inpatient and outpatient hospital contacts during the follow-up. By using hospital episode diagnoses until year 2000, we calculated a modified Charlson comorbidity index, which was utilized as surrogate for overall comorbidity and short-term risk of dying.

The information on CRPC treatment was obtained from electronic patient record system of Tampere University Hospital and included all initiation and ending dates during 2001 to 2020, type of treatment and cause of discontinuation of treatment.

### Statistical analysis

Risk of three different outcomes were examined by statin use: the risk of initiating the first-line treatment for CRPC, the risk of initiating second- or third-line treatment for CRPC and the risk for a given CRPC treatment to end due to inefficacy.

Cox proportional hazards regression was used to estimate hazard ratios (HR) and 95% confidence intervals (95% CI) for the risk of initiation of CRPC treatment (Table 3). Statin use status was regarded as a time-dependent variable, which was allowed to change on a yearly basis. The follow-up started at the initiation of ADT and ended at initiation of first-line treatment for CRPC or other closure dates of follow-up, whichever occurred first.

Logistic regression was used to estimate the odds ratios for the risk of second- and third-line CRPC treatment by statin use status after the initiation of previous CRPC treatment line (Table 4). The follow-up in these analyses started at initiation of CRPC treatment and ended at initiation of the next CRPC treatment line.

The risk of a given CRPC treatment to discontinue due to inefficacy was estimated using Cox proportional hazards regression (Table 5). Statin use status was considered as a fixed variable based on statin use status on the year of initiation of treatment and follow-up lasted for the duration of current treatment line (Tables 4 and 5).

We used adjustment for age at diagnosis, EAU risk group and Charlson comorbidity index in analyses. Statin use was considered as a time-dependent variable in analysis assessing risk of initiating CRPC treatment and as a fixed variable (statin use on initiation year of CRPC treatment) in all other analyses (risk of initiation of CRPC treatment and risk of treatment to end due to inefficacy). When statins’ effect on risk of next CRPC treatment line initiation and discontinuation of current CRPC treatment line due to inefficacy was examined, patients were divided as statin users and nonusers at the year of initiation of CRPC treatment and status was updated for each treatment line separately.

## Results

### Population characteristics

Of the 1,169 men, who received androgen deprivation therapy, 615 (52.6%) had used statins during the follow-up. During the median follow-up of 7.5 and 5.7 years, there were 99 prostate cancer deaths among statin users and 150 among non-users. In comparison, there were 289 (47.0%) and 340 (61.4%) overall deaths among statin users and non-users. Use of co-medications, i.e., antidiabetics, antihypertensive drugs, NSAIDs and aspirin was clearly more common among statin users (Table 1).

**Table 1:** Baseline characteristics, a cohort of prostate cancer patients treated with androgen deprivation therapy.

	Statin use during the follow-up		Statin use at the time of initiation of treatment for CRPC	
	None	Any	None	Any
N of men	554	615	111	50
N of PCa deaths	150 (27.1%)	99 (16.1%)	66 (59.5%)	28 (56.0%)
N of overall deaths	340 (61.4%)	289 (47.0%)	71 (64.0%)	34 (68.0%)
Median (IQR) follow-up time (years) after ADT initiation until CRPC	5.7 (2.5–9.6)	7.5 (4.4–11.8)	NA	NA
Mean age at PCa diagnosis (years)	69.7	70.2	68.4	68.7
Mean age at ADT initiation	71.1	71.6	69.8	67.3
BMI; median (IQR)*	26.9 (24.6–30.2)	26.3 (24.5–28.5)	24.4 (22.8–30.0)	NA
Median Charlson comorbidity index	2	2	2	2
Tumour stage at diagnosis				
T1-2	334 (60.3%)	417 (67.8%)	48 (43.2%)	22 (44.0%)
T3-4	220 (39.7%)	198 (32.2%)	63 (56.8%)	28 (56.0%)
Tumour Gleason grade				
6 or lower	163 (29.4%)	212 (34.5%)	18 (16.2%)	13 (26.0%)
7	175 (31.6%)	215 (35.0%)	34 (30.6%)	16 (32.0%)
8 to 10	196 (35.4%)	174 (28.3%)	58 (52.3%)	21 (42.0%)
Metastatic disease at diagnosis (M1)	102 (18.4%)	65 (10.6%)	36 (32.4%)	13 (26.0%)
PSA level at diagnosis				
20 or less	364 (65.7%)	435 (70.7%)	60 (54.1%)	31 (62.0%)
Above 20	166 (30.0%)	151 (24.6%)	47 (42.3%)	18 (36.0%)
Unknown	24 (4.3%)	29 (4.7%)	4 (3.6%)	1 (2.0%)
Choice of primary treatment				
Active surveillance	38 (6.9%)	44 (7.2%)	2 (1.8%)	1 (2.0%)
Radical prostatectomy	65 (11.7%)	60 (9.8%)	20 (18.0%)	9 (18.0%)
Radical radiotherapy	47 (8.5%)	65 (10.6%)	7 (6.3%)	8 (16.0%)
LHRH	298 (53.8%)	356 (57.9%)	56 (50.4%)	23 (46.0%)
Other	106 (19.1%)	90 (14.6%)	26 (23.4%)	9 (18.0%)
PSA relapse	206 (37.2%)	198 (32.2%)	102 (91.9%)	47 (94.0%)
EAU risk group				
Low-risk	98 (17.7%)	119 (19.3%)	10 (9.0%)	4 (8.0%)
Intermediate-risk	170 (30.7%)	228 (37.1%)	23 (20.7%)	16 (32.0%)
High-risk	286 (51.6%)	268 (43.6%)	78 (70.3%)	30 (60.0%)
Use of other medication				
Andiabetic drugs	63 (11.4%)	190 (30.9%)	9 (8.1%)	12 (24.0%)
Antihypertensive drugs	352 (63.5%)	528 (85.9%)	65 (58.6%)	42 (84.0%)
NSAIDs	456 (82.3%)	530 (86.2%)	96 (86.5%)	46 (92.0%)
Aspirin	54 (9.7%)	135 (22.0%)	6 (5.4%)	9 (18.0%)
Type of ADT (categories not mutually exclusive)				
GnRH agonist/antagonist	451 (81.4%)	520 (84.6%)	99 (89.2%)	45 (90.0%)
Antiandrogens	292 (52.7%)	296 (48.1%)	102 (91.9%)	45 (90.0%)
Orchiectomy	47 (8.5%)	33 (5.4%)	8 (7.2%)	5 (10.0%)
Radiation therapy				
None	356 (64.3%)	348 (56.6%)	83 (74.8%)	28 (56.0%)
Yes	198 (35.7%)	267 (43.4%)	34 (30.6%)	16 (32.0%)
Employment status				
Employed	60 (10.8%)	53 (8.6%)	16 (14.4%)	7 (14.0%)
Unemployed	12 (2.2%)	14 (2.3%)	3 (2.7%)	1 (2.0%)
Retired	475 (85.7%)	544 (88.5%)	92 (82.9%)	42 (84.0%)
Unknown	7 (1.3%)	4 (0.7%)	0	0
Marital status				
Single/Divorced/Widow	189 (34.1%)	141 (22.9%)	27 (24.3%)	10 (20.0%)
Married/Registered partnership	365 (65.9%)	474 (77.1%)	84 (75.7%)	40 (80.0%)

*information available for 94 patients.

During the follow-up, in total 161 men initiated first-line CRPC treatment (Table 2). Of those, 50 PCa patients used statins during the year of treatment initiation. Median duration of first-line CRPC treatment did not differ markedly by statin use (158 days for statin users and 146 for non-users). In total, 91 men ended up initiating second-line of CRPC treatment. Reflecting treatment and reimbursement practice in the early 2000s and late 1990s, first-line treatment was most often docetaxel. As a second treatment line, abiraterone or enzalutamide were most common. In addition to these medications, there were also some patients participating in research projects and using different drug combinations. Patients who initiated treatment for CRPC were more likely to die from PCa and have more aggressive PCa (higher PSA, stage, grade and EAU risk group) at diagnosis compared to the whole ADT cohort.

**Table 2:** Baseline characteristics, a cohort of prostate cancer patients treated with androgen deprivation therapy who initiated castration-resistant prostate cancer (CRPC) treatment during the follow-up.

	Statin use at the initiation of CRPC treatment	
	No	Yes
First line CRPC treatment		
Median duration of first line CRPC treatment (IQR)	146 (86–266)	158 (90–255)
N of men	111	50
N of PCa deaths	66 (59.5%)	28 (56.0%)
N of overall deaths	71 (64.0%)	34 (68.0%)
Docetaxel	47 (42.3%)	20 (40.0%)
Enzalutamide	22 (19.8%)	11 (22.0%)
Abiraterone	8 (7.2%)	3 (6.0%)
Other	34 (30.6%)	16 (32.0%)
Second line CRPC treatment		
Median duration of second line CRPC treatment (IQR)	144 (83–318)	130 (70–182)
N of men	71	20
N of PCa deaths	45 (63.3%)	11 (55.0%)
N of overall deaths	48 (67.6%)	12 (60.0%)
Docetaxel	21 (33.3%)	4 (18.2%)
Enzalutamide	6 (9.5%)	5 (22.7%)
Abiraterone	22 (34.9%)	8 (36.4%)
Other	13 (20.6%)	3 (13.6%)
Cabazitaxel	1 (1.6%)	2 (9.1%)

### Risk of initiating first line CRPC treatment by statin use

The median duration from ADT initiation to first-line CRPC treatment was 4.1 years for statin non-users (111/68.9%) and 6.0 years for statin users (50/31.1%), respectively. Statin use was associated with a decreased risk of initiation of CRPC treatment (HR 0.68; 95% CI 0.47–0.97) (Table 3). The risk decrease remained similar in both age-adjusted and multivariable-adjusted analyses. When risk of initiating CRPC treatment was assessed by statin use intensity tertiles, no trend was observed between the groups and the risk decrease remained statistically significant only in the 2^nd^ tertile. When prevalent statin users were excluded, results remained similar but no statistically significant association was observed (HR 0.71; 95% CI 0.43–1.18).

**Table 3:** Risk of initiation of treatment for castration-resistant prostate cancer (CRPC) by statin use in a cohort of prostate cancer patients initially managed with androgen deprivation therapy.

	Risk of initiation of CRPC treatment by statin use	
	Age-adjusted	Multivariable-adjusted*
Non-users	Reference	Reference
Statin users	0.66 (0.46–0.95)	0.68 (0.47–0.97)
Intensity of statin use		
1st tertile	0.68 (0.25–1.84)	0.70 (0.26–1.89)
2nd tertile	0.46 (0.25–0.85)	0.46 (0.25–0.86)
3rd tertile	0.85 (0.50–1.43)	0.82 (0.49–1.39)

*Adjusted for age at diagnosis, EAU risk group and Charlson comorbidity index.

### Risk of initiating second and third line CRPC treatment by statin use

Although, the point estimates of HRs were consistently below 1 in all analyses, no statistically significant difference in the risk of initiating second (OR 0.63; 95% CI 0.32–1.26) or third-line (OR 0.57; 95% CI 0.19–1.72) CRPC treatment was observed. In subgroup analyses stratified by median amount of DDDs used at the initiation year of CRPC treatment line, risk modification was not observed, either (Table 4).

**Table 4:** Risk of initiation of sequential lines of castration-resistant prostate cancer (CRPC) treatment by statin use at the initiation of previous CRPC treatment line in the cohort of Finnish prostate cancer patients treated with androgen deprivation therapy.

	Risk of initiation of second line CRPC treatment by statin use at the initiation of first line treatment		Risk of initiation of third line CRPC treatment by statin use at the initiation of second line treatment	
	Age-adjusted	Multivariable-adjusted*	Age-adjusted	Multivariable-adjusted*
Non-users	Ref	Ref	Ref	Ref
Statin users	0.60 (0.30–1.17)	0.63 (0.32–1.26)	0.59 (0.20–1.73)	0.57 (0.19–1.72)
Statin DDDs below median*	0.74 (0.29–1.88)	0.81 (0.31–2.10)	0.18 (0.02–1.56)	0.17 (0.02–1.47)
Statin DDDs above median*	0.76 (0.31–1.91)	0.76 (0.30–1.92)	1.14 (0.32–4.09)	1.15 (0.30–4.34)

*Median DDDs for the first line CRPC treatment line was 261,3 and for second line CRPC treatment line 200,0.

### Risk of CRPC treatment to end due to inefficacy

We did not observe any meaningful differences by statin use in the risk of discontinuation of CRPC treatment due to inefficacy (Table 5). Hazard ratios for first (HR 1.04; 95% CI 0.58–1.88) and second-line treatment (HR 0.99; 95% CI 0.38–2.59) were similar. In subgroup analysis stratified by choice of CRPC treatment, no clear differences between groups were observed, either. When the risk of death from all causes was examined, statin use was associated with slightly increased risk (HR 1.33; 95% CI 0.89–1.99) compared to non-use at the initiation of CRPC treatment.

**Table 5:** Risk of castration-resistant prostate cancer (CRPC) treatment to end due to inefficacy by statin use at the time of treatment line initiation in a cohort of prostate cancer patients treated with androgen deprivation therapy.

	Risk of first line CRPC treatment to end as ineffective by statin use status		Risk of second line CRPC treatment to end as ineffective by statin use status	
	Age-adjusted	Multivariable-adjusted*	Age-adjusted	Multivariable-adjusted*
All treatment choices	1.18 (0.68–2.06)	1.04 (0.58–1.88)	0.83 (0.34–2.00)	0.99 (0.38–2.59)
Enzalutamide or abiraterone	1.16 (0.43–3.12)	0.63 (0.16–2.49)	2.97 (0.89–9.95)	2.52 (0.72–8.76)
Docetaxel or kabazitaxel	1.04 (0.38–2.83)	1.02 (0.37–2.80)	0.43 (0.05–3.77)	1.65 (0.17–16.32)
Other	1.05 (0.41–2.71)	1.42 (0.50–3.98)	0.55 (0.07–4.57)	0.46 (0.05–4.22)

*Adjusted for age at diagnosis, EAU risk group and Charlson comorbidity index.

### The duration of CRPC treatment line by statin use status

**Figure 2:**
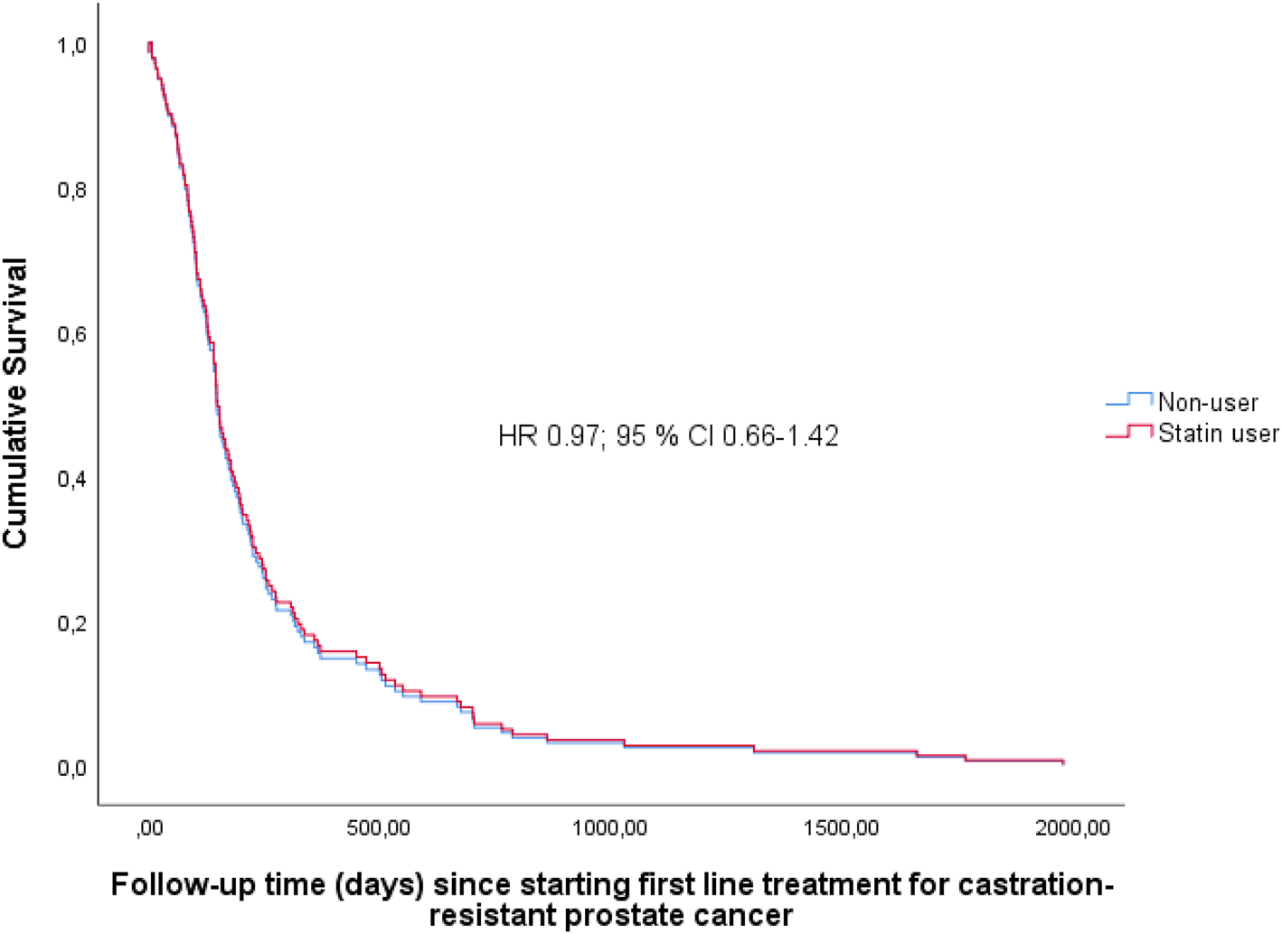
Cumulative follow-up time in days until discontinuation of first line treatment for castration-resistant prostate cancer by statin use.

**Figure 3:**
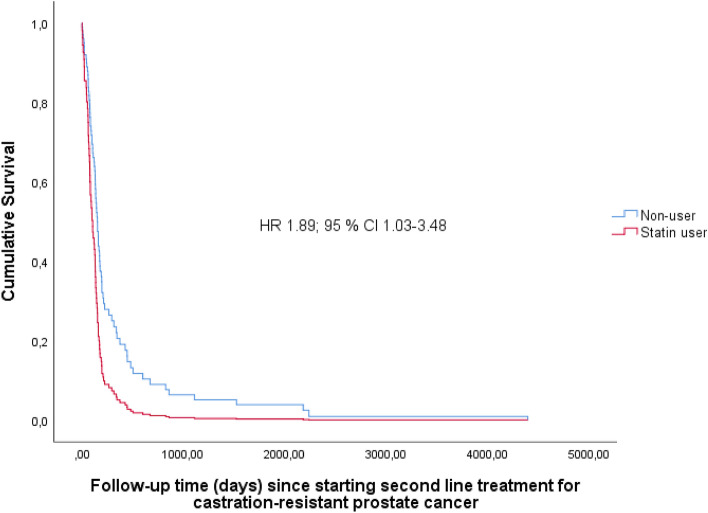
Cumulative follow-up time in days until discontinuation of second line treatment for castration-resistant prostate cancer by statin use.

The duration of first-line CRPC treatment was not affected by statin use status (HR 0.97; 95% CI 0.66–1.42) (Fig. 2). However, we observed statistically significantly longer duration of the second treatment line among non-users of statins (HR 1.89; 95% CI 1.03–3.48, median duration 144 days for non-users and 130 days for users) (Fig. 3).

## Discussion

Statin use among men receiving ADT for PCa was associated with a lower risk of initiating CRPC treatment. This is consistent with previous findings suggesting that statin use may enhance the therapeutic effect of ADT. In contrast, statin use was not associated with risk of initiating second- or third-line CRPC treatment or treatment discontinuation for inefficacy. Contrary to our initial hypothesis, statin users were statistically significantly more likely to have shorter duration of second-line CRPC treatment than non-users.

Three recent meta-analyses have shown statins to be associated with improved overall survival in prostate cancer patients treated with ADT^13,21,27^. Two of those studies also found statins to be linked with better prostate cancer-specific survival^13,21^. Especially, the statin use after initiation of ADT has provided most consistent results showing statins to improve prognosis in patients with advanced PCa. In our analysis, the follow-up started at the initiation of ADT and only statin use occurring at the year or after the initiation of ADT was taken into account with time-dependent statin use variables that were allowed to change on a yearly basis. This enabled us to manage immortal time bias which is known to potentially cause results favoring medication users in pharmacoepidemiologic studies^28^.

A couple of previous in vitro studies have found statins to have additive inhibitory effect on prostate cancer cell growth in combination with docetaxel^17–20^. To our knowledge, there are no published epidemiological studies assessing specifically the effect of docetaxel or other taxans in combination with statins on prostate cancer prognosis. Our study cohort consisted of men whose primary CRPC treatment was mostly (41.6%) docetaxel reflecting the treatment guidance and reimbursement practice in the beginning of twenty-first century and thus we were able to analyze statins’ potential benefit in combination with docetaxel. In subgroup analyses, we did not observe statistically significant difference in the risk of first or second line taxan treatment line to discontinue as ineffective between statin users and non-users.

Previously, some epidemiological studies have found statins to improve prognosis in castration-resistant prostate cancer treated with enzalutamide or abiraterone^21–24^. However, at least one study showing no benefit from statins in combination with abiraterone exists^29^. In our cohort, we did not observe differences by statin use status in the risk of enzalutamide or abiraterone treatment to discontinue due to inefficacy. However, our cohort size in regard of subgroup analyses was relatively small and minute differences between groups were not detectable in this analysis. Nonetheless, in our analysis of duration of second line CRPC treatment which consisted mainly of abiraterone or enzalutamide, we observed statistically significantly longer second treatment line duration in men not treated with concurrent statins. This finding that is not in line with previous results might be explained by increased risk of side effects or poorer toleration of treatment among statin users due to e.g. comorbidities or poor performance status which are known to be linked both to statin use status and treatment-associated adverse effects^30,31^. Additionally, our analysis examining the risk of treatment line to end due to inefficacy differs from previous studies by outcome measure as survival, per se, was not in scope.

The durations of treatment lines for CRPC were relatively short and therefore we were forced to use mostly fixed statin use variables in these analyses predisposing us to healthy-user bias as discontinuation of statins becomes more likely with shorter life-expectancy. As no statistically significant differences despite this potential source of bias favoring statin users were observed, this is suggestive that statins do not have marked benefit in this late phase of prostate cancer treatment. In addition, healthy user bias would probably not pose a major problem in this situation because PCa patients initiating treatment for CRPC still may have years of life remaining and therefore discontinuation of statin use is unlikely to happen that early. Considering the current evidence, we suppose the optimal timing of statin use in relation to prostate cancer treatment to be concurrently with androgen deprivation therapy.

The drug reimbursement practice in Finland has changed since the start of follow-up as at that time enzalutamide or abiraterone purchases were reimbursed only for patients previously treated with docetaxel. The current treatment guidance by European Association of Urology recommends the initiation of abiraterone or enzalutamide increasingly in the earlier phase of disease limiting the generalizability of these results for the present^32^. The participants of FinRSPC study were followed-up during 1996–2017 which represents the era when androgen receptor signaling inhibitors (ARSI) were initiated less frequently and mostly for the second line treatment of CRPC. Due to that fact the number of patients treated with ARSIs remains relatively low limiting detection of possible differences in subgroup analysis.

We were able to analyze the association between statin use and the risk of developing castration resistant prostate cancer and the outcomes of CRPC treatment by statin use in our population-based cohort of 1,169 men initiating ADT during the follow-up. The comprehensive and reliable data provided by national databases and electronic patient records enabled us to conduct a multi-variable analysis assessing the risk of ending to CRPC by using time-dependent statin use variables and adjustment for patient age, EAU risk group and Charlson comorbidity index. The precise information on the durations of CRPC treatment lines allowed us to compare the outcomes of first and second line CRPC treatment by statin use status at the initiation of each treatment lines. However, we did not have information on behavioral factors such as smoking, diet, use of health services and physical activity which might represent potential confounding factors.

Based on the results of this study and previous studies, we believe statins to be of most benefit for the treatment of hormone-sensitive prostate cancer in combination with ADT. It can be assumed that statin-induced decrease in circulating cholesterol, which is a precursor for steroid hormone synthesis, eventually leads to decrease in androgen biosynthesis. Therefore, of medications currently on the market for the treatment of prostate cancer, statins’ anticancer effects probably resemble most abiraterone which reduces androgen production by blocking enzyme CYP17. Similarly to abiraterone, statin use may provide survival benefit especially when used in the hormone-sensitive phase.

Our results support previous epidemiological studies showing statin use may improve survival in concurrent use with ADT and suggest better PCa-specific survival among statin users to be explained by longer time to development of castration-resistant prostate cancer. Considering the main results of this study and the results of previous studies assessing the prognosis of PCa patients by statin use, we suggest the optimal time window for statin use to be concurrently with androgen deprivation therapy. Therefore, future trials assessing the benefit of statin use in PCa prognosis should focus especially on that period when plenty of quality life years can still be saved.

## Conclusions

In our population-based cohort consisting of 1,169 men initiating ADT during the follow-up, inverse association between statin use and the risk of developing CRPC was observed. This finding is in line with previous studies detecting statins might be linked to improved prostate cancer prognosis among ADT treated patients. In our cohort, we did not observe statistically significant improvement by statin use in survival of CRPC patients. These results are suggestive that optimal timing of repurposing statins on prostate cancer treatment is concurrently with androgen deprivation therapy.

## Data Availability

The data that support the findings of this study are available on request from the corresponding author. The data are not publicly available due to their containing information that could compromise the privacy of research participants.
